# On the rewards to international investing: a safe haven currency perspective

**DOI:** 10.1186/s41937-017-0005-8

**Published:** 2018-06-19

**Authors:** Jean-Pierre Danthine, Samuel Danthine

**Affiliations:** 1 Paris School of Economics and CEPR, Paris, France; 2 Center of Research in Economics and Statistics - Ensai, Rennes, France

**Keywords:** Uncovered interest parity, Safe haven currency, Currency hedging, F30, F31, G11

## Abstract

The safe haven property of the Swiss franc presents a specific challenge for internationally minded Swiss-based investors. The central issue is whether the traditional under-performance of Swiss assets is made up by the secular appreciation of the Swiss franc combined with the propensity of the safe haven to strengthen in times of market stress. In this paper, we review the evidence on the terms of this challenge. We conclude that a Swiss bias in asset allocation can lead to considerable return shortfalls over the long run and that systematic currency hedging would not have been historically justified and is unlikely to be in the future. Assuming a fair amount of currency risk thus appears inevitable for long-run Swiss-based investors.

## Background

The appreciation of the Swiss franc since the start of the financial crisis has been a traumatic experience for Swiss-based investors, repeatedly turning their currency exposures into severe losses. The impact of this experience is hard to overestimate. Ten years later, broad anecdotal evidence and current account statistics suggest that the propensity of Swiss-based investors (and corporates) to assume currency risk has been durably reduced when not annihilated. With extremely low—in fact currently negative—risk-free interest rates in francs, this implies that prudent investors—notably pension funds—face a very high hurdle in order to deliver the investment returns that are counted on. Macroeconomically, the reluctance to take on currency risks has meant the end of the previously prevailing external balance of payment equilibrium whereby the current account surplus is compensated by equi-proportionate external net capital flows. As a consequence, the Swiss National Bank (SNB) has been forced into substituting public capital flows to the missing private ones and in so doing has accumulated a balance sheet exceeding 100% of GDP as of this writing.

The sudden and massive appreciation of the franc during the crisis is one of three factors rendering particularly challenging the natural desire of residents of a small country such as Switzerland of reaping the benefits of international portfolio diversification. The second determinant of the specific challenge facing Swiss franc-based investors is the secular tendency of the franc to appreciate in nominal and also, according to Baltensperger and Kugler (2016), in real terms against the major alternative currencies. This trend presents a significant hurdle when the proceeds of international investments are converted back into the home currency. Acting as a countervailing force, however, is the persistent negative interest differential—sometimes termed the Swiss interest island—that has characterized the returns on CHF-denominated assets (relative to those observed in other major markets) almost since the first world war but notably since the end of the Bretton Woods period (Baltensperger and Kugler 2016).

Using data from 1980 to 2003, that is before the Great Financial Crisis, Kugler and Weder (2004) came to the conclusion that the negative interest differential outweighed the tendency of the franc to appreciate so that an unhedged rolled over 3-month position in dollars, UK sterling, and euros converted in Swiss francs delivered superior returns to a pure Swiss franc investment in 3-month securities. They thus observed that uncovered interest parity (UIP) had not been validated over their period of observation. This result is consistent with more general observations made in the carry-trade literature (see Menkhoff et al. (2012) and the extensive literature cited therein) but the interest rate island, that is, the persistence of the sign of the interest differential in the case of the CHF and, as a consequence, the length of the investment holding periods contemplated by (Kugler and Weder 2004) made their results distinctive. (Kugler and Weder 2004) further observed the over-performance of an unhedged investment in foreign currency 10-year bond portfolios while a Swiss franc equity investment had delivered a superior return compared to equity portfolios in each of the three foreign currencies after conversion into francs.

Taken at face value, the Kugler and Weder (2004) results could lead to the conclusion that the current reluctance of Swiss-based investors to take on currency risk is misguided, possibly due to a behavioral bias resulting from the size of the trauma mentioned above and its historical proximity. Such a sweeping conclusion would be premature, however, because their sample did not include a crisis period which is precisely when the safe haven characteristics that are the counterpart of the interest island manifest themselves in a way that is meaningful for long-run investors.

In this paper, we return to this issue with the benefit of a severe crisis being part of our period of observation. The Swiss negative interest rate differential has been a puzzle for a long time (and it is the focus of Kugler and Weder’s inquiry). We believe the additional period of observation permits reaching a coherent and logical explanation for this phenomenon. The recent literature on safe haven currencies emphasize the high frequency reactions of the CHF and the yen notably in times of surges in market volatility (Ranaldo and Söderlind 2010). We complete this view here by adopting a long-run perspective. A safe haven asset is also one which provides insurance against rare but severe shocks with a long-lasting impact. This insurance must have a price in the form of offering lower returns in normal times (thus making it a natural funding currency for carry traders), or else, the asset in question would be a dominating asset^1^. It is not surprising therefore that, in the case of the CHF, uncovered interest parity does not hold, possibly for long, crisis-free time periods. But this normal return deficit may be made up in crisis times when the safe haven characteristics of the currency manifest itself with a vengeance. We will see that this view is consistent with the observations over the last 36 years and highlight the ensuing challenges facing Swiss-based investors.

Our interest for the current problematic of investors based in a small country leads us to push the inquiry one step further and make a comparison between hedged and unhedged returns, taking the cost of hedging into account. Our goal is to address what we perceive as the currently dominant practice of Swiss-based investors which is to form highly internationalized portfolios, if only for lack of opportunities given the limited size of the Swiss capital markets, but systematically hedge the corresponding currency risks. We observe that the question of whether it pays to hedge or not is directly related to the existence of significant and long-lasting deviations from UIP. In other words, in a world of long-lasting deviations from interest parity, currency hedging is not the free lunch; it is sometimes made to be and often hoped for at the current juncture. But the crisis behavior of the safe haven currency may render unhedged international investment portfolios (or a permanent carry trade position for that matter) excessively risky. We suggest that a selective hedging strategy based on the strength of the franc has good chances of being successful and provide an example of a simple-minded strategy that would have dominated a systematic hedge over the past 36 years.

## Uncovered interest parity

As a starter, Fig. 1 depicts the evolution of the bilateral exchange rates between the CHF and the euro, US dollar, and the pound sterling since the beginning of our sample in 1980 until the end of 2016.^2^ It powerfully feeds the fear that unhedged investments in foreign assets by Swiss-based investors are prohibitive: the trend-like appreciation of the CHF gives the appearance of a formidable obstacle for earning a positive return after conversion of one’s investment into francs.

Figure 2 provides a radically different perspective. It displays the cumulative return obtained over the period by rolling over 3-month money market (MM) portfolios in dollars, euros/DM, and UK sterling as opposed to a similar portfolio in CHF.^3^ It illustrates the dilemma presented Swiss franc investors: the returns in the three alternative currencies are significantly more attractive than those that can be expected from a direct investment in franc. With the power of compounding, the difference over a period as long as the one adopted here can be huge.

**Fig. 3:**
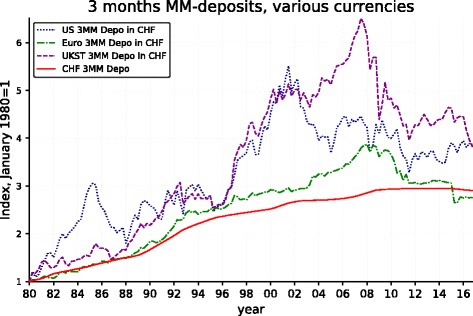
Cumulative return on 3-month MM deposits after conversion into CHF

Naturally, a good deal of the nominal return differences shown in Fig. 2 must be accounted for by inflation differences between the corresponding countries, with Switzerland exhibiting lower average rates of inflation. Simultaneously, these inflation differentials have much to do with the evolution of the exchanges rates observed in Fig. 1. But real exchange rates (i.e., exchange rates corrected for the inflation differentials) are not constant, deviations from purchasing power parity can be long lasting and, as already mentioned, the possibility of a secular *real* appreciation of the franc cannot be excluded. The critical issue for a Swiss-based investor is therefore whether, over the investment horizon, the positive return differentials on investments in foreign currencies more than compensate for the nominal appreciation of the CHF. Figure 3 shows that this has largely been the case for the dollar and the sterling, less spectacularly so for the euro. One sees that the dollar and sterling 3-month portfolios would have obtained a cumulative return significantly in excess of the similar investment in CHF, even after conversion into the Swiss currency. Moreover, one observes that the cumulative excess returns (over the CHF portfolio) can take temporarily extremely large values. This was also the case for the Euro-DM portfolio up until 2008. However, the very strong CHF appreciation with respect to the euro that coincided with the advent of the Great Financial Crisis has progressively annihilated the accumulated return differential which even turned negative after the jump of the CHF coinciding with the abolition of the minimum exchange rate between the CHF and the euro.

Table 1 summarizes the average return differences with the CHF portfolio for various sub-periods. Line 2 shows the underperformance of the CHF portfolio before the crisis. Line 4 displays the strong reversal observed during the crisis especially for the euro and pound portfolios. Line 1 shows that, over the entire period, the return difference averages approx. 1.5% per year for the dollar and the pound portfolios while approximate parity is achieved with the euro.^4^

**Fig. 4:**
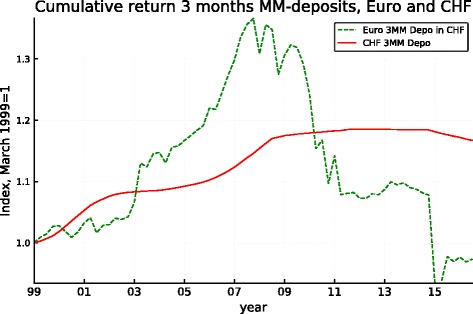
Cumulative performance of the euro and CHF portfolios since the advent of the euro

Given the hybrid nature of the comparison with the euro portfolio, it makes sense to look at the euro portfolio since the advent of the common currency. We do this in Fig. 4 which delivers a clear message: the euro portfolio strongly over-performed in “peacetime" but this was followed, with the advent of the financial crisis, by a reversal in favor of the CHF portfolio, leading to almost exactly 8 years of superiority of the euro portfolio followed by 8 years of under-performance.

These observations confirm that ex-post deviations from uncovered interest parity can be long lasting. They also show that in normal times, the return differential pays off in the sense that, over the years, it more than compensates for the nominal appreciation of the franc. Moreover, the cumulative return advantage over long periods can be substantial. In crisis times, however, the appreciation of the safe haven currency combined with the compressed interest differentials at the Zero-Lower-Bound leads to reversals where investments in the safe haven currency delivers superior returns, as should be expected. Note that in the case of the dollar, the weakness of the early 2000s has been as significant as the financial crisis itself. This is no doubt a reflection of the fact that the dollar also possesses safe haven characteristics. As shown in Fig. 4, the reversal has been dramatic in the case of the euro, plausibly because the euro area has been at the center of the crisis, so that the (smaller) return advantage cumulated until the advent of the crisis has been eliminated. Viewed from this angle, the hypothesis of uncovered interest parity appears validated. This validation, however, comes with the modulo that one may have to wait for a very severe crisis before observing the equalization of returns (and, as for all low-frequency events, such an event may not materialize in the course of a finite length sample). The reversal has been in full force for the UK sterling as well, but the return advantage cumulated until 2007 was too large to have been wiped out by the advent of the crisis. The situation is similar in the case of the dollar. Here again, the return advantage cumulated until approximately 2000 was so substantial that it was not annihilated by the subsequent under-performance of the dollar portfolio.

For later reference, note that if we denote $\hat {r}_{t}$ as the interest rate on 3-month deposit in CHF, $\hat {r}^{*}_{t}$ as the corresponding rate for a 3-month deposit in one of our three alternative currencies, and $\hat {r}^{*\text {CH}}_{t}$ as the foreign return after conversion into CHF, the return difference in Table 1 can be written

1 $$ \begin{aligned} \hat{r}^{*\text{CH}}_{t} - \hat{r}_{t}&=\left[ \left(1+\hat{r}^{*}_{t}\right) \frac{E_{t+1}}{E_{t} }-1\right] -\hat{r}_{t} \\& =\left(1+\hat{r}^{*}_{t}\right)\left[ \frac{E_{t+1}}{E_{t} }-\frac{1+\hat{r}_{t}}{1+\hat{r}^{*}_{t}}\right], \end{aligned}  $$

where *E*_*t*_ is the spot exchange rate between the CHF and the relevant currency.

## Bond portfolios

We now take a look at the relative performance of 10-year bond portfolios in the four currencies under consideration. Adopting the perspective of a long-run investor, we compute returns for holding periods of 1 year for bond and equity (next section) portfolios. The long-lived deviations from uncovered interest parity displayed in the last section are sure to have some influence on the longer maturity assets but differences in the dynamics of term premia and inflation risk premia across currencies are also plausible. This is notably the case because of the well-known link between inflation levels and volatility suggesting that inflation risk premia and their dynamics could differ between currencies.

Table 2 shows that until the advent of the crisis, differences in return between 10-year bond portfolios in dollar, UK, and euro (bund), on the one hand, and a 10-year CHF portfolio, on the other, were substantial with an order of magnitude of 4, 6, and 2% per annum, respectively. Worth noting is how these return differences were compressed during the crisis (but not annihilated), in particular in the case of the dollar and the sterling. Since this was precisely the time where the safe haven currency appreciated, one can expect that the nominal return differences in that sub-period were no longer sufficient to compensate investors for the change in the value of the Swiss franc.

**Table 2:** Return differentials on 10-year bond portfolios in local currency

Period	Euro	USD	UKST
1980–2016	2.10	3.06	4.74
1980–2007	2.16	3.88	5.61
1980–2003	2.25	4.45	6.30
2008–2016	1.93	0.58	2.15

Note: One year holding period return on 10-year benchmark government bond portfolios in various currencies after conversion in CHF net of the return on a comparable CHF portfolio

Table 3 confirms this intuition by displaying average return differences after conversion into Swiss francs. The crisis time was indeed a period where the CHF portfolio offered superior return but the superiority of the CHF portfolio over the last 8 years has not been sufficient to compensate for the return deficit over the preceding period 27 years. Even after taking the crisis period and the strong appreciation of the CHF into account, substantially higher average returns are recorded over the entire period most markedly for the US and UK bond portfolios with excess returns of 1.6 and 1.8% per annum, respectively.^5^

Here again, it is worth inquiring whether the hybrid status of the euro comparison before 1999 biases our results. As in the case of the 3-month money market portfolios, the euro portfolio clearly dominates the CHF portfolio for the first 8 years of existence of the euro (with an average yearly over-performance of 2.19%) while the reverse is true (under-performance of 3% yearly) for the next 8 years as shown in Table 3.

## Equity portfolios

Let us now have a look at equity portfolios. Tables 4 and 5 compare equity returns in foreign currencies and in Swiss franc. Table 5 confirms the observation of Kugler and Weder (2004) that the positive return differences observed in the fixed income portfolios are not uniformly duplicated when it comes to equity, notably not in euro and in sterling. While the foreign currency equity portfolios uniformly dominate the CHF equity portfolio in local currencies, the Swiss equity portfolio has over-performed the euro and sterling equity portfolios after conversion into Swiss francs. There is substantial volatility across period, however, and the performance of the franc during the crisis appears to be the dominating factor for these two currencies. By contrast, the strong performance of the dollar equity portfolio since the trough of the crisis is the critical factor explaining the superiority of the dollar equity portfolio over the entire period.^6^

The main message of this and the two preceding sections may be that while a Swiss bias in international asset allocation can be understood as the product of fear (notably following a severe crisis), such a bias may prove very costly over the long run. It is true that over a horizon of a few years, the evolution of the exchange rate can be dominant and have devastating consequences on unhedged portfolio returns. While the dollar weakness of the early 2000s has been significant, the exchange rate impact is felt most critically during a crisis period when international diversification from a safe haven currency base appears very disadvantageous. This no doubt explains the current proclivity of Swiss investors to “remain at home.”

But the data also show that the cumulative return differences in normal times can be very large, i.e., the price paid for indulging a Swiss investment bias, measured in terms of the return shortfall over long horizons, can be extremely high. And the general positive return difference in favor of international assets means that despite the tendency of the franc to appreciate over time, the benefits of international diversification can, in normal times, be obtained at little or no cost for a Swiss investor with a long view.^7^ Admittedly, in the long run, we are all dead but, much before, we turn pensioners and this renders this observation highly relevant!

The temptation to believe that one could have the cake and eat it too may be present, however. After all, a Swiss-based investor could hope to take advantage of the positive return differences highlighted in Tables 3 and 5 while at the same time systematically hedging currency risks. We address this issue in the next section.

## Should Swiss investors hedge currency risks?

Assuming a Swiss-based investor is convinced by the message of the previous sections and wishes a significant fraction of her portfolio to be invested in international bond and equity portfolios, should she be advised to hedge the corresponding currency risks taking hedging costs into account?^8^

To answer this question, we will approximate the hedging cost with the yearly equivalent of the difference between the 3-month return in alternative currency and the 3-month return in CHF. That is, we postulate, un-controversially until recently, that covered interest parity (CIP) holds but more controversially that there are no other transaction costs in covering a bond or an equity portfolio over the long run.^9^ By doing so, we certainly underestimate the hedging cost and our results should be viewed in this light.

Together, with the observed negative return differential over short horizons, CIP implies that hedging currency risk entails a significant cost for a Swiss investor. The cost of hedging long bond or equity portfolios could nevertheless be justified by large exchange rate moves. To investigate this issue, we adopt once again a long-run perspective, i.e., we do not focus on the reduction of short-run volatility offered by currency hedging. We are more concerned with the impact of hedging on returns, that is, on the question whether over time the protection against large exchange rate movements justifies the hedging costs.

Before looking at the results, it is illuminating to have an analytical examination of the returns under consideration. Assuming covered interest parity, we can measure the ratio of the Forward (*F*) to the Spot exchange (*E*) as

2 $$\begin{array}{*{20}l} \frac{F_{t,t+1}}{E_{t}}=\frac{1+\hat{r}_{t}}{1+\hat{r}^{*}_{t}}. \end{array} $$

Defining *I*_*t*_ recursively as $ I_{t+1}=(1+r^{*}_{t})I_{t}, I_{1}=1$, we compute the return on the hedged portfolios as

3 $$\begin{array}{*{20}l} r^{*h}_{t}&=\frac{I_{t}F_{t,t+1}+\left(I_{t+1}-I_{t}\right)E_{t+1}-I_{t}E_{t}}{I_{t}E_{t}} \end{array} $$

4 $$\begin{array}{*{20}l} &=\frac{F_{t,t+1}}{E_{t}}+\frac{I_{t+1}E_{t+1}}{I_{t}E_{t}}-\frac{I_{t}E_{t+1}}{I_{t}E_{t}}-\frac{I_{t}E_{t}}{I_{t}E_{t}} \end{array} $$

5 $$\begin{array}{*{20}l} &= r^{*}_{t}\frac{E_{t+1}}{E_{t}}+\frac{F_{t,t+1}}{E_{t}}-1\text{ or, alternatively,} \end{array} $$

6 $$\begin{array}{*{20}l} & r^{*CH}_{t}+\frac{F_{t,t+1}}{E_{t}}-\frac{E_{t+1}}{E_{t}}, \end{array} $$

where $r^{*\text {CH}}=\frac {I_{t+1}E_{t+1}-I_{t}E_{t}}{I_{t}E_{t}}$ is the foreign return after conversion into CHF.

These return differences are not dependent on the nature of the underlying asset. They apply equally for the bond and for the equity portfolios.

Observe that using Eq. (2) into Eq. (6), one obtains

7 $$\begin{array}{*{20}l} r^{*\text{CH}}_{t} - r^{*h}_{t} = \frac{E_{t+1}}{E_{t}}-\frac{1+\hat{r}_{t}}{1+\hat{r}^{*}_{t}}. \end{array} $$

Comparing now this last equation with Eq. (1), one sees that the issue of whether it pays to hedge is in fact another version of the question of whether UIP is validated ex-post:

8 $$\begin{array}{*{20}l} r^{*\text{CH}}_{t} - r^{*h}_{t} = \frac{\hat{r}^{*\text{CH}}_{t} - \hat{r}_{t}}{1+\hat{r}^{*}_{t}}. \end{array} $$

The long-run deviations from UIP observed in the “Uncovered interest parity” section therefore equally imply the presence of long time periods where hedging does not pay. Yet, during other, shorter, “crisis” periods, exchange rate moves swamp the interest differential, thus justifying incurring the hedging cost.

The differences in average returns between unhedged and systematically hedged portfolios are recorded in Table 6 where we have added to the three main sub-periods considered so far a computation of the average return differences for all the 5-year sub-periods in our sample. These results confirm the observations made in the “Uncovered interest parity” section showing not surprisingly that hedging foreign currency portfolios has paid in crisis times (line 4) but not when one considers the entire period of observation, except in the case of the euro. In other words, focusing on the dollar and the sterling and adopting a long-run perspective, the cost of hedging has not been covered by the average appreciation of the franc including the crisis-induced strengthening registered since 2007. Limiting ourselves to the period of existence of the euro, one would have been well advised to leave the euro portfolio unhedged for the first 8 years of the existence of the euro (superior performance of 2.12% for the unhedged portfolio from 1999 to 2007) while of course, the reverse has been true since the crisis as already noted.

**Table 6:** Average yearly teturn differences—unhedged minus hedged portfolios

Period	Euro	US	UK
1980–2016	−0.77	1.53	1.27
1980–2007	0.18	2.24	3.21
1980–2003	−0.30	2.50	3.04
2008–2016	−3.76	−0.71	−4.77
1980–1985	−3.25	8.68	1.51
1985–1990	−0.30	−8.19	3.22
1990–1995	−2.09	−4.37	−2.43
1995–2000	0.48	7.92	7.78
2000–2005	1.14	−1.09	1.21
2005–2010	−2.06	−1.06	−3.39
2010–2016	−3.71	0.01	−3.06

Note: Differences of the mean quarterly returns on unhedged and hedged portfolios, annualized

**Fig. 5:**
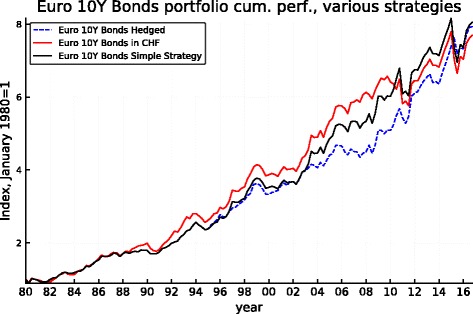
Euro 10-year bond cumulative performance: unhedged, hedged, and simple strategy

**Fig. 6:**
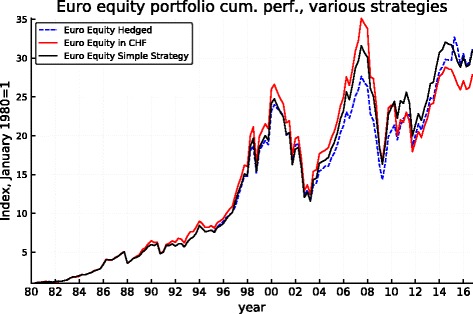
Euro equity cumulative performance: unhedged, hedged, and simple strategy

**Fig. 7:**
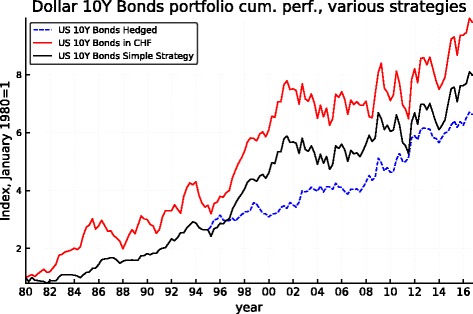
Dollar 10-year bond cumulative performance: unhedged, hedged, and simple strategy

**Fig. 8:**
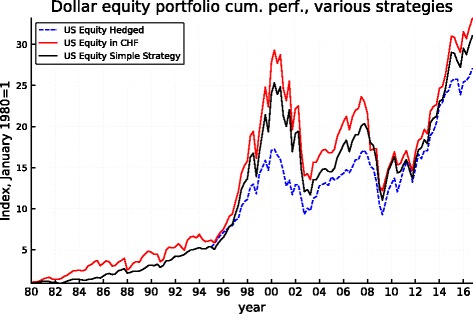
Dollar equity cumulative performance: unhedged, hedged, and simple strategy

**Fig. 9:**
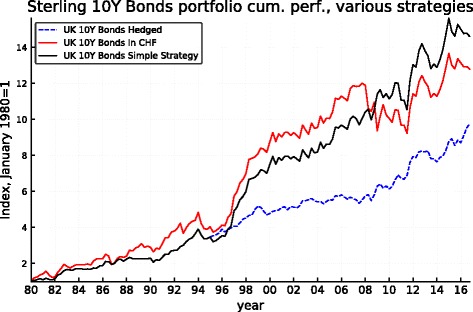
Sterling 10-year bond cumulative performance: unhedged, hedged, and simple strategy

**Fig. 10:**
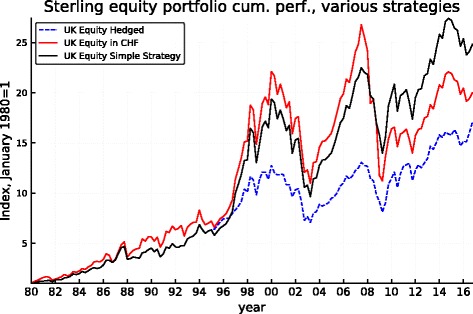
Sterling equity cumulative performance: unhedged, hedged, and simple strategy

Figures 5, 6, 7, 8, 9, and 10 record the cumulative performance of hedged and unhedged 10-year bond and equity portfolios in the three alternative currencies under consideration. These figures, whose content does not deviate from the message of Fig. 3, show that over the 36-year period, the cumulative performance of unhedged portfolios has exceeded the performance of hedged portfolios in dollar and in sterling while the cumulative performance of unhedged and hedged portfolios in euro has been very similar with a slight advantage for the hedged portfolios. Importantly, the graphs show clearly that systematic hedging of the foreign currency portfolios promise long sub-periods of very significant under-performance, the most dramatic ones being the period 1980–1985 and 1994–2000 in the case of the dollar (these were two periods of dollar appreciation) and 1994–2007 for the sterling (similarly one long period of strong sterling). The offsetting factor is of course the impact of a few prolonged up-moves in the Swiss franc, during the crisis of course but also associated with the dollar weakness of the early 2000s.

This configuration presents a significant challenge for Swiss franc-based investors. The long periods of very significant under-performance disqualify in our view a policy of systematic hedging of currency risks. Taking into account the additional intermediation, transaction costs associated with portfolio hedging could make a policy of systematic currency hedging prohibitive. Yet it is also a fact that the periods of sharp CHF appreciation loom large over the overall portfolio performance and they should be avoided if at all possible. All in all, these observations strongly support a policy of selective currency hedging by a long-run Swiss-based investor. This in turn requires a careful analysis of FX relationships so as to be in position to make informed decisions on the timing of the hedging decisions. Here is not the place to develop an exchange rate forecasting model and we should maintain the presumption that crises are essentially unpredictable.^10^ But we can modestly build on the return-towards-the mean property of real exchange rates over the medium to long run.

To illustrate, it might be judicious for a long-run investor to refrain from hedging when the real value of its base currency is high on a historical basis—say *x* percent above its long-run trend—and conversely to make sure a hedge is locked in when it is abnormally low—say *y* percent below trend. We illustrate a simple-minded strategy in this spirit below. Concretely, we assume that the default position is fully hedged, that the investor unlocks the hedge when the real effective exchange rate (REER) of the CHF is 10% above its historical mean, and that she reinstates the hedge when the REER falls more than 5% from its historical mean. The historical mean is computed with the data available to the decision maker at the time of her hedging decision, concretely using exchange rates from 1974 onwards (to have some prior history over which to compute the mean although starting with the 1980 exchange rate would not modify the result; the reason not to go beyond 1974 is to avoid using the compressed values of the CHF resulting from the Bretton Woods regime and its immediate removal).

One could, and should, go much further in terms of sophistication: a practically intended rather than purely illustrative implementation could focus on bilateral exchange relations and would take account of the upward tendency of the Swiss real exchange rate vis-a-vis the dollar and the pound uncovered by Baltensperger and Kugler (2016).^11^

**Fig. 11:**
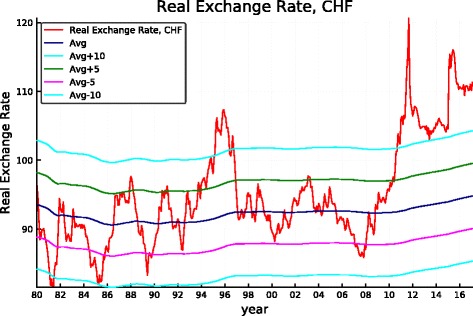
CHF real exchange rate

The robustness of such a simple strategy and its characteristics can be derived from Fig. 11. It makes clear that the application of this simple rule based on the REER of the Swiss franc leaves the portfolios unhedged starting about in mid-1994, thus permitting to benefit from the long period of superior returns of the unhedged portfolios that follows while being partly protected when the strong appreciation of the CHF started in 2007 (but the hedge is lifted during the course of 2011 leaving the portfolio unprotected thereafter). The performance of the simple-minded strategy is displayed in Figs. 5, 6, 7, 8, 9, and 10. It clearly delivers superior returns in the case of the British pound, and it beats the hedged strategies but not the unhedged portfolios in the case of the dollar, while it is tied with the hedged portfolios in the case of the euro.

## Conclusions

Switzerland’s external equilibrium is predicated on a steady stream of private (net) capital exports balancing the structural current account surplus of the country. This equilibrium has been disrupted since the Fall of 2008. In the current situation of disequilibrium, very significant public capital exports (in the form of FX interventions by the SNB) are the only protection against an even stronger CHF. The question of whether a return to the pre-crisis equilibrium is likely is of prime importance for the country. A negative answer combined with the observation that permanent interventions by the SNB must be ruled out implies a structurally much stronger CHF eroding the competitiveness of large portions of the Swiss industry until the current account surplus is eliminated.

This study has reviewed the plausibility of a return to the pre-crisis equilibrium. It has shown that the positive return differentials obtained on foreign currency investments over Swiss assets has historically provided an appropriate compensation for the currency risk attached to the CHF and has permitted generating superior cumulative returns on unhedged investments in non-Swiss assets. Selective currency hedging excluding episodes where the Swiss franc was extraordinarily strong would have generated an appreciable return boost.

While the usual word of caution “past performance is no guarantee for future returns” is appropriate, the qualitative properties of a safe haven currency highlighted in this paper should prevail in the future as they have in the past 36 years. We are thus led to the conclusion that the current situation with a very strong franc is ideal for undertaking the currency risks associated with international investments. The conditions for a return to the pre-crisis external equilibrium are fulfilled; Swiss investors were ready to put behind the trauma of the crisis-induced extraordinary appreciation of the franc and ready to hear the French saying: “La peur est mauvaise conseillère”.

## Footnotes

1 This argument is fully in line with the explanation for the high performance of carry trades given by Menkhoff et al. (2012): high-interest currencies deliver low returns in times of unexpected high volatility while low-interest currencies provide a hedge by yielding positive returns in those circumstances.

2 Our exchange rate quotes are taken from the Bank of England website. Prior to 1999, the euro is represented by a synthetic euro exchange rate calculated by geometrically weighting the bilateral exchange rates of the (then) 11 euro area countries using “internal weights” based on the country shares of extra euro area trade (Germany counting for 33%). For details, see http://www.bankofengland.co.uk/statistics/Pages/iadb/notesiadb/spot_rates.aspx

3 Annualized 3-month deposit rates are obtained from Datastream. In the case of the euro/DM deposit, the asset is a 3-month deposit in DM until the end of December 1998 with the corresponding exchange rate being the DM/CHF rate provided on the SNB website and a 3-month euro deposit after January 1999 converted with the corresponding euro/CHF exchange rate). In the next sections, we also use the 10-year benchmark government bond indices from Datastream (notably the bund converted at the DM/CHF exchange rate before 1999, at the euro/CHF rate thereafter) and the relevant MSCI equity indices also from Datastream.

4 The 1980–2003 averages enable a comparison with the results of Kugler and Weder (2004). For the 1980–2003 period, the return differentials obtained by Kugler and Weder are 0.48 (DM/euro), 2.15 (dollar), and 2.45 (pound). Our results are qualitatively similar but not identical most probably because of discrepancies in the data series used. Our tables and diagrams suggest the data volatility makes it perilous to focus on a single length sample.

**Table 1:** Return differences on 3-month deposits

Period	Euro	USD	UKST
1980–2016	−0.01	1.65	1.34
1980–2007	1.09	2.70	3.60
1980–2003	0.90	2.71	3.22
2008–2016	−3.78	−0.70	−4.80

Note: Annualized returns on 3-month deposits after conversion in CHF in excess of the return on a 3-month CHF portfolio

5 The corresponding values obtained by Kugler and Weder for the 1980–2003 period are 1.30, 4.46, and 3.55 for the DM-euro, dollar, and pound portfolios, respectively. Again, the results are qualitatively similar but not identical. A straightforward explanation for the difference is our decision to focus on 1-year holding period returns while they compute quarterly return differentials (which are annualized).

**Table 3:** Return differentials on 10-year bond portfolios after conversion in CHF

Period	Euro	USD	UKST
1980–2016	0.81	1.58	1.77
1980–2007	2.08	2.56	3.77
1980–2003	1.80	3.32	3.77
2008–2016	−3.00	−1.34	−4.23

Note: One year holding period return on 10-year benchmark government bond portfolios in various currencies after conversion in CHF net of the return on a comparable CHF portfolio

6 As before, the results of Kugler and Weder are well aligned with ours: −1.43, 0.74, and −1.03 for the euro-DM, dollar, and pound portfolios, respectively, but not identical. Here again, one source of discrepancy is our focus on 1-year holding period return. Another is that our euro equity portfolio is a true MSCI Europe index for the entire period (and not a German index) converted pre-1999 at the synthetic euro exchange rate mentioned in footnote 2.

**Table 4:** Return differentials on equity portfolios in local currency

Period	Euro	USD	UKST
1980–2016	1.19	1.40	0.97
1980–2007	1.50	0.40	0.91
1980–2003	1.65	1.55	1.62
2008–2016	0.20	4.51	1.18

Note: One year holding period return on MSCI equity indices in various currencies after conversion in CHF net of the return on a comparable CHF portfolio

**Table 5:** Return differentials on equity portfolios after conversion in CHF

Period	Euro	USD	UKST
1980–2016	−0.98	0.56	−1.39
1980–2007	0.11	0.04	−0.49
1980–2003	−0.38	1.60	−0.43
2008–2016	−4.37	2.20	−4.20

Note: One year holding period return on MSCI equity indices in various currencies after conversion in CHF net of the return on a comparable CHF portfolio

7 It is worth repeating that our goal is not to perform a full portfolio optimization where the additional diversification benefits of international investments would be tallied but rather to clarify the return dimension of such investments in the specific case of the safe haven CHF.

8 Again, the full answer would require a full portfolio optimization where the covariance between the hedged and unhedged return series and the other relevant return series would play a central role.

9 CIP was viewed as something close to a physical law until the crisis, but it has been consistently violated since. This entails a supplementary element in the cost of hedging. See Cecchetti and Schoenholtz (2016), Borio et al. (2016), and references therein.

10 The appreciation of the CHF since 2007 has, however, been a protracted move.

11 Menkhoff et al. (2017) propose adjusting the bilateral real exchange rates for key country-specific fundamentals, thus getting at a more robust measure of currency values. Their results are entirely in the spirit and fully supportive of what we are proposing although they are not specifically geared towards the specific case of a safe haven currency. Using ER data since 1970, Lambelet (2016) suggests the REER of the CHF also exhibits a deterministic upward trend. The property is less secure, however, if one omits the 1970–1980 data, a period which is marked by delayed adjustments following the end of the Bretton Woods regime. Taking a positive trend in the REER of the CHF into account would only marginally influence our simple strategy and not materially improve its performance.
